# Production of Carotenoids and Phospholipids by *Thraustochytrium* sp. in Batch and Repeated-Batch Culture

**DOI:** 10.3390/md20070416

**Published:** 2022-06-25

**Authors:** Allison Leyton, Carolina Shene, Yusuf Chisti, Juan A. Asenjo

**Affiliations:** 1 Centre for Biotechnology and Bioengineering (CeBiB), Center of Food Biotechnology and Bioseparations, BIOREN and Department of Chemical Engineering, Universidad de La Frontera, Francisco Salazar 01145, Temuco 4780000, Chile; allison.leyton@ufrontera.cl; 2 School of Engineering, Massey University, Private Bag 11 222, Palmerston North 4442, New Zealand; ychisti@hotmail.com; 3 Centre for Biotechnology and Bioengineering (CeBiB), Department of Chemical Engineering and Biotechnology, Universidad de Chile, Beauchef 851, Santiago 8370459, Chile; juasenjo@ing.uchile.cl

**Keywords:** *Thraustochytrium* sp., thraustochytrids, carotenoids, canthaxanthin, docosahexaenoic acid, phospholipids

## Abstract

The carotenogenic thraustochytrid *Thraustochytrium* sp. RT2316-16 was grown in batch and repeated-batch cultures using different feeds containing glucose, or glycerol, and yeast extract, for the production of lipids, phospholipids and carotenoids. RT2316-16 produced canthaxanthin, astaxanthin and β-carotene. The effects of biotin, ascorbic acid, light and temperature were evaluated in some of the experiments. In 2-day-old batch cultures, the combined mass percentage of eicosapentaenoic acid and docosahexaenoic acid in total lipids was between 16.5% (glycerol-based medium in the dark; biomass concentration = 4.2 ± 1.1 g L^−1^) and 42.6% (glucose-based medium under light; biomass concentration = 3.3 ± 0.1 g L^−1^), decreasing to 3.8% and 6.1%, respectively, after day 4. In repeated-batch cultures, the total lipids in the biomass increased after glucose or glycerol was fed alone, whereas the total carotenoids (168 ± 7 μg g^−1^ dry weight (DW)) and phospholipids in the biomass increased after feeding with yeast extract. The biomass with the highest content of phospholipids (28.7 ± 4.3 mg g^−1^ DW) was obtained using a feed medium formulated with glycerol, yeast extract and ascorbic acid. Glycerol was the best carbon source for the production of a biomass enriched with total lipids (467 ± 45 mg g^−1^ DW). The composition of carotenoids depended strongly on the composition of the feed. Repeated-batch cultures fed with yeast extract contained canthaxanthin as the main carotenoid, whereas in the cultures fed only with glucose, the biomass contained mainly β-carotene.

## 1. Introduction

Thraustochytrids are heterotrophic protists found in diverse aquatic habitats [1]. Certain marine thraustochytrids are of interest as they produce nutritionally important long-chain polyunsaturated fatty acids (PUFA) including docosahexaenoic acid (DHA, C22:6n-3) and eicosapentaenoic acid (EPA, C20:5n-3). This, in combination with the high levels of total lipids in their biomass, makes them particularly attractive for use in aquaculture feeds and as sources of PUFA for human nutrition [1].

DHA in thraustochytrids occurs mainly in triacylglycerols (TAG) and phospholipids. Phospholipids are components of cell membranes, and their content in the biomass and composition depends on the phase of the cell cycle. In the phospholipids of *Aurantiochytrium limacinum* F26-b, DHA represent about 50% of total fatty acids [2]. Major phospholipids in this strain were 16:0–DHA-phosphatidylcholine (35%) and 16:0–DHA-phosphatidylethanolamine (20%) [3]. The fatty acid composition of phospholipids in *Schizochytrium limacinum* SR21 changed dramatically with the age of the culture: in the 1-day-old cells, DHA composed 61.7% of the total fatty acids in phospholipids, decreasing to 8.7% in cells harvested from a 10-day-old culture [4]. In some thraustochytrids, the phospholipid fraction in the total lipids remained relatively stable with culture age. For example, in *Schizochytrium* sp. A-2, the phospholipid content remained at around 20% of the total lipids after 48 h [5]. Similarly, in *Aurantiochytrium mangrovei* Sk-02, phospholipids constituted ~18% of total lipid, irrespective of the incubation temperature (which varied from 12 to 35 °C) [6]. In a thraustochytrid-like microorganism, the TAG content in the biomass decreased with a simultaneous increase in the phospholipid content after the cells grown in a glucose-rich medium were transferred to a glucose deficient medium [7]. This shift in lipid composition (from TAG to phospholipids) has also been reported in *Schizochytrium* sp. [8,9].

DHA in phospholipids has attracted attention because it may be an alternative to delivering DHA to the brain [10,11] as compared with DHA in TAG. As phospholipids are produced during the production of DHA-enriched TAG in thraustochytrids, it is of interest to understand how the culture conditions, especially the medium composition, might affect the phospholipid content in the biomass and in total lipids.

In addition to producing DHA-rich lipids, some thraustochytrids also produce carotenoids such as astaxanthin, β-carotene, canthaxanthin, lutein, zeaxanthin and fucoxanthin [1,12]. The specific function of carotenoids in thraustochytrids is unknown. However, because of their structural features, carotenoids can modify the structure, properties and stability of cell membranes, where they are commonly located [13]. Carotenoids are known for their antioxidant activity, a property that depends on the molecular structure of the carotenoid and is influenced by interactions with lipids and proteins. Natural carotenoids, mainly from photosynthetic microalgae, represent a small fraction of a market that is dominated by chemically synthesized products [14]. Although natural carotenoids are more expensive, they are in demand for use in cosmetics, pharmaceuticals and nutraceuticals. Carotenoid contents in the biomass of photosynthetic microalgae is relatively high; however, the productivity of microalgae cultures is generally low, as the maximum biomass concentration in a photoautotrophic culture does not typically exceed 5 g L^−1^ because of limited light penetration. Compared to photosynthetic microalgae, cultures of some heterotrophically grown thraustochytrids rapidly attain high biomass concentrations [8,9].

The present work focused on establishing the effects of culture conditions (nutrients in the culture medium, temperature and illumination) on the production of lipids (total lipids and phospholipid) and total carotenoids by *Thraustochytrium* sp. RT2316-16, an Antarctic thraustochytrid capable of producing β-carotene and canthaxanthin [15]. The microorganism was grown in batch and repeated-batch cultures. The latter involved one or two feedings with different feed formulations to determine the effect on total lipids and total carotenoids in the biomass. The objective of these experiments was to determine if the production of phospholipids and carotenoids in the biomass requires different culture conditions. In some of the experiments, the effect of biotin and ascorbic acid was evaluated. Biotin is a prosthetic group in biotin-dependent carboxylases [16] that catalyze key reactions in gluconeogenesis (pyruvate carboxylase), amino acid catabolism (propionyl-CoA carboxylase, methylcrotonyl-CoA carboxylase) and fatty acid synthesis (acetyl-CoA carboxylase). Ascorbic acid is an electron donor and thus a reducing agent with the potential for decreasing the effects of oxidative stress in aerobic culture. Positive effects of ascorbic acid on the growth and DHA production by *Schizochytrium* sp. have been reported [17,18]. The results were used to identify strategies for enhancing the production of phospholipids and carotenoids in repeated-batch cultures of RT2316-16.

## 2. Results

### 2.1. Batch Culture

**Figure 1:**
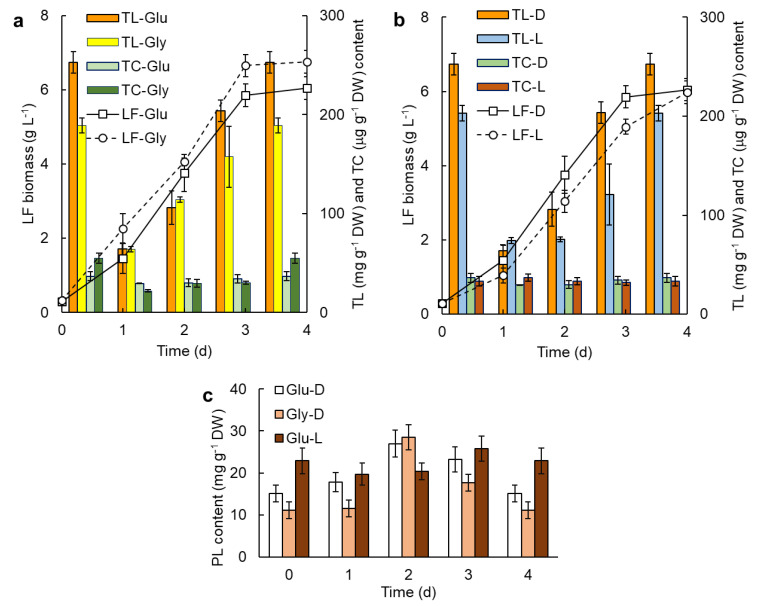
(**a**) Effect of the carbon source (glucose, Glu; glycerol, Gly) on the concentration of lipid-free (LF) biomass (symbols), the total lipids (TL) in the biomass and the total carotenoids (TC) in the biomass produced in the dark. (**b**) Effect of illumination (light, L; dark, D) on the concentration of LF biomass in the culture broth (symbols), the TL in the biomass and TC in the biomass produced using glucose as the carbon source. (**c**) Phospholipid (PL) content in the biomass grown using glucose (Glu) or glycerol (Gly) in the dark (D) or Glu in light (L). The culture temperature was always 15 °C.

Effects of different carbon sources (glucose, glycerol) and illumination (light or dark), on the behavior of 4-day batch cultures are shown in Figure 1. Data are shown for the growth of lipid-free (LF) biomass, the total lipids (TL) in the biomass, the total carotenoids (TC) in the biomass, and the content of phospholipid (PL) in the biomass.

In these experiments (Figure 1), more than 90% of the initial nitrogen, quantified as total amino acids, had been consumed by day 4 (data in Supplemental Material), but only 49% of the initial glycerol and 58% of the glucose were consumed (data in Supplemental Material). The onset of the stationary phase on day 3 (Figure 1a) was ascribed to the exhaustion of the amino acids required for the growth of lipid-free biomass. After 4 days, the total lipids in the biomass grown on glucose was 34% higher than in the biomass grown on glycerol (Figure 1a), presumably because more glucose had been consumed. In contrast to total lipids, the total carotenoids in glycerol-grown biomass (day 4) were around 50% higher than in the biomass grown on glucose (Figure 1a). The substantial increase (~80% increase) in total carotenoids from day 3 to day 4 coincided with nitrogen exhaustion and the onset of the stationary phase (Figure 1a). Light had no significant effect (*p* > 0.05) on the final concentration of lipid-free biomass or its total carotenoids content (Figure 1b), although a slight but significant (*p* < 0.05) effect of light on the final total lipids in the biomass (203 ± 8 mg g^−1^ dry weight (DW) under light; 253 ± 11 mg g^−1^ DW in the dark) was observed (Figure 1b). In view of its insignificant effect on parameters of interest in glucose-based media, the effect of light was not further studied.

RT2316-16 produced mainly canthaxanthin, astaxanthin and β-carotene (Table 1). Canthaxanthin in 4-day-grown biomass was on average 13% greater in dark growth compared to growth under light (Table 1).

The total carotenoids in the biomass varied with time, in a similar way to total lipids (Figure 1a,b). In all data shown in Figure 1, the total lipids in the biomass declined to a minimum value by day 1 of incubation. This initial decrease was followed by a progressive accumulation of lipids until harvest on day 4.

The phospholipid content in the biomass ranged from around 10 mg g^−1^ DW to a maximum of around 28 mg g^−1^ DW (Figure 1c). The peak of phospholipids during dark growth on glucose and glycerol (Figure 1c) coincided with the time (day 2, Figure 1c) at which the total lipid and total carotenoids in the biomass began to increase (Figure 1a,b) and did coincide with the mid-exponential growth (day 2, Figure 1a,b).

### 2.2. Repeated-Batch Cultures Fed Once

**Figure 2:**
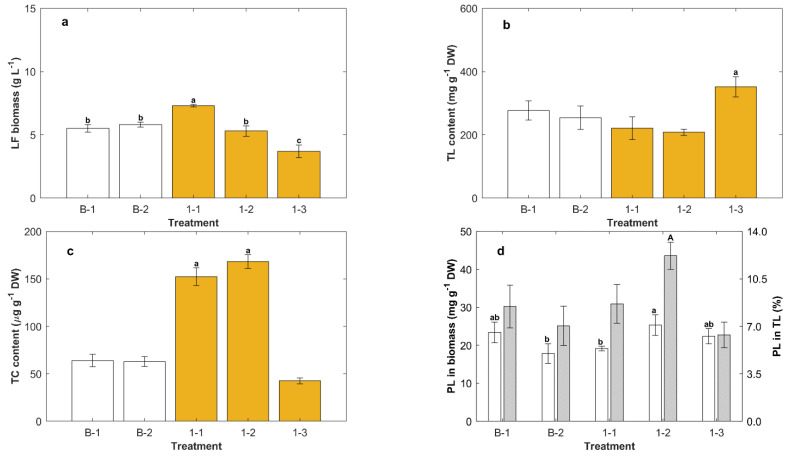
Concentration of the lipid-free (LF) biomass (**a**), the total lipid (TL) in the biomass (**b**), the total carotenoids (TC) in the biomass (**c**), the phospholipid (PL) in the biomass (**d**, white bars), and PL in the total lipid (**d**, grey bars). The biomass was grown in batch (B) culture without biotin (B-1) and with biotin (B-2). The repeated-batch cultures without biotin were fed with the CM (1-1), with only the yeast extract (1-2), or with only glucose (1-3). Different letters above bars within a graph indicate significant differences (*p* < 0.05).

After a batch phase that lasted 6 days to allow complete consumption of glucose, in separate experiments, the cultures were fed once on day 7 to increase the concentration of nutrients (glucose and yeast extract) to the same level as in the control medium (CM), i.e., glucose 20 g L^−1^ and yeast extract 6 g L^−1^, or the concentration of yeast extract to 6 g L^−1^, or the concentration of glucose to 20 g L^−1^. The fed volume (50 mL) reduced the biomass concentration to 2/3 by dilution at the end of the batch phase. The results are shown in Figure 2. Data obtained at the end of batch cultures in CM with and without biotin are also shown (Treatments B-1, B-2; Figure 2).

In the batch phase, biotin had no significant effect (*p* > 0.05) on the concentration of the lipid-free biomass (Figure 2a), the total lipids in the biomass (Figure 2b), or the total carotenoids in the biomass (Figure 2c). Four days after the CM concentrate was fed, the lipid-free biomass concentration increased by 30% (Figure 2a). If only the yeast extract was fed, the concentration of lipid-free biomass 4 days after the feeding was not significantly affected (*p* > 0.05) compared to the concentration before the feeding (Figure 2a).

If only concentrated glucose was fed, the concentration of the lipid-free biomass on day 4 was reduced to around 67% of the concentration before the feeding (Figure 2a). Thus, the lipid-free biomass concentration increased after feeding only if the complete medium was fed. Feeding with only yeast extract and only glucose did not support growth, as either the carbon source (i.e., the culture fed with only yeast extract) or the nitrogen source (i.e., the culture fed with only glucose) was insufficient to support growth. The lower lipid-free biomass concentration in Treatments 1-3 (Figure 2a) was partly a consequence of dilution and partly a result of cells dying and lysing in the absence of a nutritionally complete medium.

The quantity of total lipids in the biomass was significantly (*p* < 0.05) affected by the composition of the feed (Figure 2b). The biomass fed only with glucose had a 27% higher total lipids content (352 ± 42 mg g^−1^ DW) compared to biomass produced at the end of the batch phase (277 ± 40 mg g^−1^ DW) (Figure 2b). Feeding only with yeast extract reduced the total lipids in the biomass to 208 ± 10 mg g^−1^ DW, a 25% reduction compared to biomass from the batch phase. Similarly, feeding with concentrated CM reduced the total lipids content to 221 ± 36 mg g^−1^ DW, a 20% reduction compared to biomass from the batch phase.

Although RT2316-16 accumulated lipids during cell growth (Figure 1) in the absence of a nitrogen limitation, lipid production was enhanced by the exhaustion of nitrogen as long as the medium had available glucose. If only yeast extract was fed, the carbon supply was insufficient to support growth and the cells metabolized the stored lipids.

Feeding with only yeast extract, or the CM concentrate, significantly (*p* < 0.05) raised the total carotenoids in the biomass (a 2.6-fold rise with yeast extract; a 2.4-fold rise with CM concentrate), compared to the case before the feeding (63.9 ± 6.5 μg g^−1^ DW; Treatment B1, Figure 2c). In addition, the feed composition affected the predominant type of carotenoid produced: canthaxanthin was the major carotenoid in biomass fed with yeast extract, whereas the glucose-fed biomass contained mainly β-carotene (Table 1).

The feed composition significantly (*p* > 0.05) affected the phospholipid content of the biomass and the phospholipid proportion in total lipids (Figure 2d). Biomass with the highest level of phospholipids (25.3 ± 2.7 mg g^−1^ DW) was obtained by feeding concentrated yeast extract (Treatment 1-2, Figure 2d). This biomass had the highest fraction of phospholipids (12.2%) in the total lipids.

As confirmed in Figure 2, repeated-batch (or fed-batch) operation could be used to increase the final biomass concentration and also to modify the composition of the biomass in terms of its total lipids, total carotenoids, and phospholipids. In view of these results, both the feed composition and the frequency of feeding had a clear potential to be optimized, for example, to produce biomass high in total lipids, or high in total carotenoids. Therefore, a two-step feeding strategy with different feed compositions was explored.

### 2.3. Effect of the Carbon Source in Repeated-Batch Cultures Fed Twice

**Figure 3:**
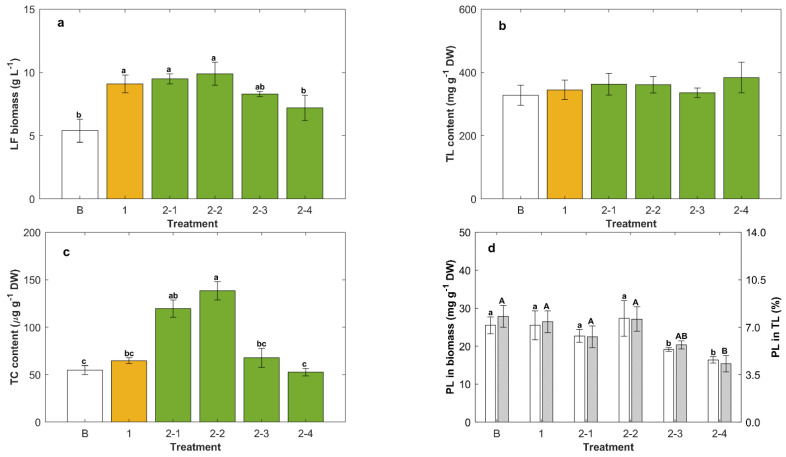
Effects of feeding treatments on lipid-free (LF) biomass (**a**), total lipid (TL) in biomass (**b**), total carotenoids (TC) in biomass (**c**), phospholipids (PL) in biomass (**d**, white bars), and PL in total lipid (**d**, grey bars). Treatments: batch (B) culture in control medium (CM); after the first feeding with a concentrated CM (1); after the second feeding with a concentrated CM (2-1); after the second feeding with a concentrated CM and ascorbic acid (2-2); after the second feeding with CM2 (2-3); and after the second feeding with glucose only (2-4). Different letters above bars within a graph indicate significant differences (*p* < 0.05).

In experiments with two feeding steps, the effect of ascorbic acid was tested in the feed at a final concentration of 2 g L^−1^. The relevant data are shown in Figure 3.

In experiments with a second feeding (50 mL), the data (Figure 3) were measured 5 days after feeding with either concentrated CM (with and without ascorbic acid at 2 g L^−1^) or with only glucose. In some cases, the second feed contained both glucose and yeast extract, so that after feeding, the culture attained 20 g glucose L^−1^ and 3 g yeast extract L^−1^ (CM2 composition). In these experiments, the biomass concentration diluted ¾ after the second feed. For comparison, the data for the batch phase (6 days after inoculation, Treatment B) and for the culture with a single feeding (5 days after the first feeding and just before the second feeding, Treatment 1) with a concentrated CM are also shown (Figure 3).

The culture fed (second feeding) with concentrated CM mixed with ascorbic acid provided the highest concentration of lipid-free biomass (9.9 ± 0.7 g L^−1^; Figure 3a), but this biomass concentration was not significantly different (*p* > 0.05) compared to the concentration reached after the second feeding with only the concentrated CM. Therefore, ascorbic acid, at least at the concentration of 2 g L^−1^, was of no consequence in terms of the final concentration of the lipid-free biomass. Feeding with CM2, or only with glucose, actually reduced the final lipid-free biomass concentration relative to the values before the feeding (Treatments 2-3 and 2-4; Figure 3a).

A second feeding, as well as the composition of this feed, had no significant effect (*p* > 0.05) on total lipids in the biomass (Figure 3b), and the average total lipids level in the twice-fed biomass was 361 mg g^−1^ DW, nearly the same as in the biomass fed once (345 ± 31 mg g^−1^ DW). In contrast with this, the total carotenoids content in the biomass was significantly affected (*p* < 0.05) by the composition of the second feed (Figure 3c): total carotenoids were the highest (138 ± 10 μg g^−1^ DW) in the biomass fed (second feeding) with CM mixed with ascorbic acid (Figure 3c). If the second feed comprised only glucose (Treatment 2-4, Figure 3c), or the composition CM2 (Treatment 2-3, Figure 3c), the total carotenoids level in the biomass was no different (*p* > 0.05) from the level in the biomass obtained either at the end of the batch operation, or after a single feeding.

If the second feed supplied only glucose, or the composition CM2, the resulting biomass had the lowest levels of phospholipids (between 16 ± 1 and 19 ± 1 mg g^−1^ DW; Figure 3d). Total lipids with the least phospholipids (17.1 ± 2.3%) were produced if only glucose was used for the second feed. These results showed that the feeding conditions that promoted the growth of lipid-free biomass also promoted production of phospholipids (Figure 3a,d).

**Figure 4:**
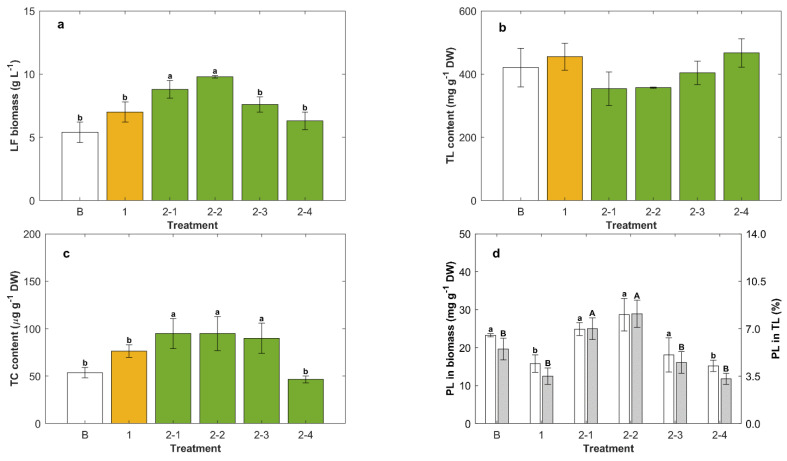
Effects of feeding treatments on lipid-free (LF) biomass (**a**), total lipid (TL) in biomass (**b**), total carotenoids (TC) in biomass (**c**), phospholipids (PL) in biomass (**d**, white bars), and PL in total lipid (**d**, grey bars). Treatments: batch (B) culture in glycerol medium CM*; after the first feeding with a concentrated CM* (1); after the second feeding with a concentrated CM* (2-1); after the second feeding with a concentrated CM* and ascorbic acid (2-2); after the second feeding with CM2* (2-3); and after the second feeding with glycerol only (2-4). Different letters above bars within a graph indicate significant differences (*p* < 0.05).

As in batch cultures, the carbon source, whether glucose or glycerol, significantly affected the final contentof the total carotenoids in the biomass (Figure 1a), single-feeding and two-feedings experiments, equivalent to those shown in Figure 3, were conducted with the modified medium CM* (i.e., CM with the glucose replaced by glycerol as the carbon source). For these experiments, the formulation CM* was used for inoculum preparation, the batch operation, and the first feed. The results are shown in Figure 4.

The concentration of lipid-free biomass was increased by 40% if the second feed with a concentrated CM* was supplemented with ascorbic acid (Treatment 2-2, Figure 4a) compared to the concentration just before the second feeding (Treatment 1, Figure 4a). The contribution of ascorbic acid to the increase of the concentration of lipid-free biomass was estimated as 12% (Treatments 2-1 and 2-2, Figure 4a).

On average, the biomass grown with glycerol had a lipid content (Figure 4b) higher than the biomass grown with glucose (Figure 3b). The highest level of total lipids (467 ± 45 mg g^−1^ DW) was produced if only glycerol was used for the second feeding (Treatments 2-4, Figure 4b); this total lipid level was 20% higher than for the biomass grown on glucose under otherwise equivalent culture conditions (Figure 3b). Once nitrogen had been exhausted after the first feeding, feeding with glycerol enhanced the accumulation of total lipids in the biomass (Treatment 2-4, Figure 4b). This was because glycerol provided both carbon and energy for continued synthesis of lipids, but there was no nitrogen to support biomass growth (Figure 4a). If the second feed contained yeast extract (Treatments 2-2 and 2-3; Figure 4b), the resulting biomass had lower total lipids (average of 350 mg g^−1^ DW) than the biomass that was fed only once with the concentrated CM* (455 ± 43 mg g^−1^ DW; Treatment 1, Figure 4b).

The presence of the yeast extract in the second feed enhanced the total carotenoids in the biomass by 20% (Treatments 2-2 and 2-3; Figure 4c) compared to the biomass fed once with the concentrated CM* (Treatment 1; Figure 4c), but the Treatment 2-2 biomass (Figure 4c) had a total carotenoids level that was only 69% of the level in the biomass that was grown on glucose in otherwise identical conditions (Treatment 2-2, Figure 3c).

In experiments involving glycerol as the carbon source, biomass with the highest level of phospholipids (28.7 ± 4.3 mg g^−1^ DW) was produced if the second feed contained ascorbic acid (Treatment 2-2; Figure 4d), but this phospholipids level was not significantly different (*p* > 0.05) compared to that in the biomass grown in a batch operation (Treatment B, Figure 4d). The phospholipids content of total lipids ranged between 3.3 ± 0.4% (second feeding with glycerol only; Treatment 2-4, Figure 4d) and 8.1 ± 1.2% (second feeding with concentrated CM* mixed with ascorbic acid; Treatment 2-2, Figure 4d).

### 2.4. Effect of Temperature in Repeated-Batch Cultures

**Figure 5:**
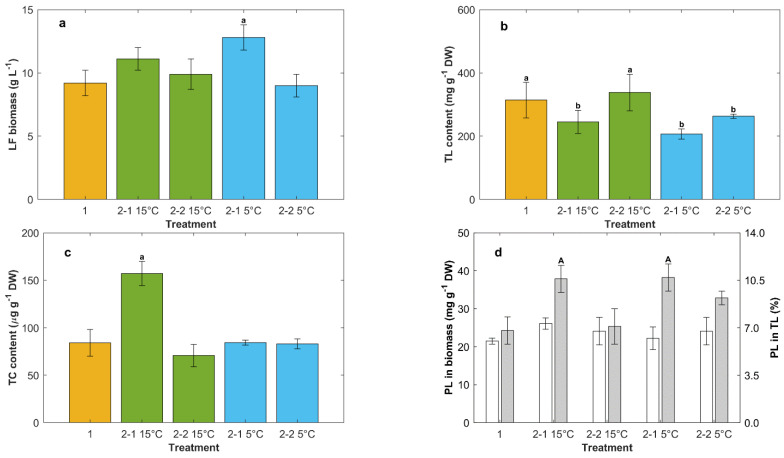
Effect of feed composition (second feeding) and the incubation temperature (5 °C, 15 °C) after the second feeding on: lipid-free (LF) biomass (**a**), total lipid (TL) in biomass (**b**), total carotenoids (TC) in biomass (**c**), phospholipids (PL) in biomass (**d**, white bars), and PL in total lipid (**d**, grey bars). Feed composition: fed once with a concentrated control medium CM (1); second feeding with a concentrated CM3 to raise the concentration of glucose (30 g L^−1^) and yeast extract (6 g L^−1^) (2-1); second feeding with only glucose (to raise the concentration to 30 g L^−1^) (2-2). Prior to the second feeding, the culture temperature was 15 °C in all cases. Different letters above bars within a graph indicate significant differences (*p* < 0.05).

In some experiments, the incubation temperature was changed from the normal 15 °C to 5 °C after the second feeding with feeds that either raised the concentrations of glucose and yeast extract to 30 g L^−1^ and 6 g L^−1^ (i.e., CM3 composition), respectively, or raised only the glucose concentration to 30 g L^−1^ (Figure 5).

The highest concentration of lipid-free biomass (12.8 ± 1.9 g L^−1^) was obtained after the second feeding of CM3 in combination with incubation at 5 °C (Treatment 2-1 5 °C, Figure 5a). This concentration of lipid-free biomass was 40% higher than the concentration obtained after the first feeding with the concentrated CM (Treatment 1, Figure 5a) and 10% higher than the concentration obtained if the identically fed culture was incubated at 15 °C (Treatment 2-1 15 °C, Figure 5a).

The effect of the composition of the second feed on the total lipids in the biomass was significant (*p* < 0.05) when the incubation temperature was 15 °C (Treatments 2-1 15 °C and 2-2 15 °C, Figure 5b). In contrast, the feed composition at the lower incubation temperature did not significantly (*p* > 0.05) affect the content of total lipids in the biomass (Treatments 2-1, 5 °C, and 2-2, 5 °C; Figure 5b).

Temperature affected the total carotenoids in the biomass. The total carotenoids content was the highest (157 ± 13 μg g^−1^ DW) for the incubation temperature of 15 °C in combination with second feeding with CM3. This maximum carotenoids content was 1.9-fold higher (Treatment 2-1, 15 °C, Figure 5c) than the total carotenoids in the biomass obtained after first feeding with the concentrated CM (Treatment 1, Figure 5c). Incubation at 5 °C reduced the growth rate compared to incubation at 15 °C, although this effect is not seen in Figure 5a because the harvest times were different (harvest was 6 days after the second feeding for cultures incubated at 15 °C, and it was 10 days after the second feeding for the cultures incubated at 5 °C). The longer incubation period at the lower temperature was to allow a similar level of glucose consumption (~5 g L^−1^) in the cultures incubated at different temperatures. The results might suggest that if the feed contained yeast extract, the total carotenoids in the biomass were higher for the cells grown at a higher specific growth rate, i.e., at the higher incubation temperature. For otherwise identical conditions, incubation at 5 °C reduced the total lipids in the biomass compared to incubation at 15 °C (Figure 5b).

The incubation temperature and the composition of the second feed had no significant effects (*p* > 0.05) on phospholipids content in the biomass (Figure 5d). The percentage of phospholipids in total lipids in the biomass harvested after the second feeding with CM3 was the highest (10.6%; Figure 5d) and did not depend on the incubation temperature.

### 2.5. Effect of Cold Storage after Lipid Synthesis in Repeated-Batch Cultures Fed Twice

**Figure 6:**
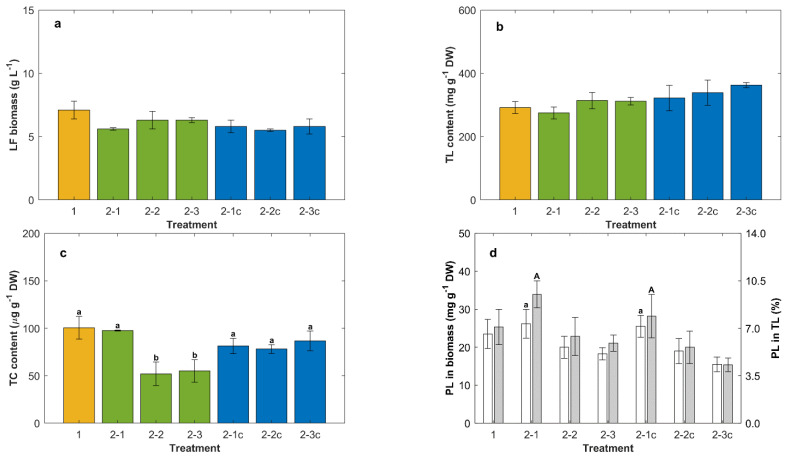
Effect of the glucose concentration in second feeding on lipid-free (LF) biomass (**a**), total lipid (TL) in biomass (**b**), total carotenoids (TC) in biomass (**c**), phospholipids (PL) in biomass (**d**, white bars), and PL in total lipid (**d**, grey bars). All cultures were grown at 15 °C in the control medium (CM) and fed once with concentrated CM (1). Glucose concentration attained after the second feed (g L^−1^): 20 (2-1), 30 (2-2) and 40 (2-3). The cultures 2-1c, 2-2c and 2-3c were stored for 24 h at 4 °C after the residual glucose concentration decreased to ~5 g L^−1^. Different letters above bars within a graph indicate significant differences (*p* < 0.05).

In a further set of experiments, the effect of a 24 h storage at 4 °C on the total lipids, the total carotenoids and the phospholipids in the biomass harvested after the second feeding with glucose only was examined. The objective was to determine if lipid synthesis occurred during storage. In different experiments, the glucose concentration attained after the second feeding was 20, 30 or 40 g L^−1^. In all cases, the biomass was harvested after the glucose concentration had declined to ~5 g L^−1^. This occurred on day 6 if the glucose concentration after the feeding was 20 g L^−1^. The harvest was on day 7 if the glucose concentration after the feeding was 30 g L^−1^, and if the concentration after feeding was 40 g L^−1^, the harvest occurred on day 8. The relevant data are shown in Figure 6.

The concentration of lipid-free biomass and the total lipids in the biomass were not significantly (*p* > 0.05) affected either by the glucose concentration after the second feeding or by the subsequent 24 h storage at 4 °C (Figure 6a,b). The nitrogen limitation in cultures fed only with glucose explained a near absence of growth in lipid-free biomass, although the cultures grew sufficiently to compensate for the dilution that inevitably occurred after the feeding.

The total carotenoids in the biomass were significantly (*p* < 0.05) affected by feeding with different quantities of glucose (Treatments 2-1, 2-2 and 2-3; Figure 6c). If the glucose concentration after the feeding was ≥30 g L^−1^, the biomass had nearly 50% less total carotenoids compared to biomass fed at a glucose concentration of 20 g L^−1^ (Treatments 2-1, 2-2 and 2-3; Figure 6c). Irrespective of the glucose concentration attained after the second feeding, the 24 h storage at 4 °C after the residual glucose decreased (~5 g L^−1^) raised the total carotenoids in all biomass samples to an average of 82 μg g^−1^ DW (Treatments 2-1c, 2-2c, 2-3c; Figure 6c).

After the second feeding with glucose at its lowest concentration, the phospholipid content in the biomass increased by around 10% compared to the biomass harvested after the first feeding (Figure 6d). The total lipids in this biomass had the highest proportion of phospholipids (26 ± 7 μg g^−1^ DW) (Treatment 2-1 white and grey bars; Figure 6d). The subsequent storage at 4 °C had no significant effect (*p* > 0.05) on the phospholipids in the biomass or in the total lipids.

### 2.6. Fatty Acid Composition of Total Lipids in RT2316-16

The fatty acid composition of the total lipids extracted from RT2316-16 depended strongly on the time of harvest of the biomass (time since inoculation in batch culture) and the carbon source (glucose or glycerol) used (Table 2).

By day two, the sum of EPA and DHA in the total lipids reached values between 16.5% (glycerol, dark) and 42.6% (glucose, light), decreasing to 3.8% and 6.1%, respectively, after day 4.

The fatty acids in the total lipids of the biomass grown in repeated-batch culture after the second feeding with glucose only, or with the concentrated CM3 (Figure 5), are shown in Table 3. Data are shown for incubation at 5 °C and 15 °C after the feeding.

Both the incubation temperature after the feeding and the composition of the feed affected the fatty acids profile. The main observations (Table 3) were the following:

1. The odd-carbon fatty acids (C15:0 and C17:0) occurred (≥1.2% of TL) in the biomass grown in CM3 but not in the biomass grown on glucose.
2. The monounsaturated fatty acid (C16:1, C18:1, C24:1) content in total lipids of the CM3-fed biomass grown at 15 °C was 80% greater than in the total lipids of the biomass grown at 5 °C. However, if the feed comprised only glucose, the three noted monounsaturated fatty acids in total lipids were only 20% more at 5 °C compared to 15 °C.
3. Irrespective of the feed, γ-linoleic acid (C18:3cis6,9,12) was found only in the biomass grown at 5 °C and not in the biomass grown at 15 °C. In contrast with this, irrespective of the feed, tetracosanoic acid (C24:0) was found only in total lipids of the biomass grown at 15 °C.
4. EPA and DHA occurred in all lipids, irrespective of the composition of the feed and the incubation temperature. In all cases, substantially more DHA existed in total lipids than EPA.

## 3. Discussion

The effects of the culture operational scheme (batch, repeated-batch), the composition of the feed and the temperature on production of total lipid, phospholipids and carotenoids by RT2316-16 were characterized for possible use in the production of multiple products (lipids, carotenoids) as might occur in a thraustochytrid-based biorefinery [19]. Neutral lipids rich in DHA and EPA, phospholipids and carotenoids are some of the compounds that can be extracted from RT2316-16.

Biotin and ascorbic acid were found to have little or no effect on biomass growth and lipid synthesis. A lack of a biotin effect was possibly due to its endogenous synthesis: a search of the genome data of RT2316-16 [15] revealed the presence of most genes for the enzymes of the biotin biosynthesis pathway. The relatively minor effects of ascorbic acid on the growth of lipid-free biomass and total carotenoids in the biomass depended on the carbon source. The lack of a strong effect of ascorbic acid was intriguing and may be better understood through a future metabolome analysis of the cells.

### 3.1. Effect of Light on the Production of Lipid Compounds

Although light had no significant effect on the final concentration of lipid-free biomass and total carotenoids, and a small effect on the content of total lipids, it did affect the specific growth rate of the lipid-free biomass (Figure 1b) and both the composition of the fatty acids in total lipids (Table 2) and the carotenoids (Table 1). The enhanced β-carotene in total carotenoids may have been a result of the light negatively affecting the hydroxylation and ketolation reactions involved in converting β-carotene to canthaxanthin. Although high light is known to upregulate the expression of carotenogenic genes in cyanobacteria such as *Nostoc punctiforme* PCC 73102 to increase synthesis of canthaxanthin [20], no effect of light on the expression of specific carotenogenesis gene(s) has been reported in thraustochytrids. However, white light has been used to induce carotenoid synthesis in *Schizochytrium* sp. SH104 [21].

### 3.2. Effect of the Carbon Source on the Production of Lipids

The total lipids in the biomass grown with glucose and glycerol declined to a minimum value by day 1 of incubation (Figure 1a,b). This pattern suggests that on the transfer of inoculum to a nitrogen-rich fresh medium, the cells metabolized the accumulated lipids to produce lipid-free biomass and metabolites before de novo lipid synthesis. An alternative explanation is that the rate of lipid catabolism exceeded the rate of lipid synthesis in a nitrogen-sufficient medium.

In batch culture, glycerol proved to be a better carbon source than glucose in supporting the production of biomass rich in total carotenoids, but glucose was better for promoting the accumulation of total lipids (Figure 1a).

These differences were ascribed to differences in the metabolism of glucose and glycerol by the cells. Once taken up by the cell, glycerol is phosphorylated to glycerol-3-phosphate (G3P) in the cytosol. G3P interlinks glycolysis, lipogenesis and oxidative phosphorylation. G3P is involved in the regeneration of NAD+ (nicotinamide adenine dinucleotide) from NADH (nicotinamide adenine dinucleotide reduced form) in the cytosol via the glycerophosphate shuttle. In addition, G3P is involved in the regeneration of mitochondrial FADH2 (reduced form of flavin adenine dinucleotide, FAD) from FAD. This reaction is brought about by mitochondrial FAD-glycerol-3-phosphate dehydrogenase (mGPDH) embedded in the mitochondrial membrane and oriented in such a way that it does not require G3P to be transported into the mitochondria. As mGPDH is a source of superoxide [22], glycerol in the culture medium has the potential to stimulate the production of reactive oxygen species (ROS) that may induce membrane lipid peroxidation. A plausible cellular response is to produce carotenoids (lipid soluble ROS-quenchers) in attempts to mitigate the damage. Therefore, an enhanced activity of mGPDH in cells grown on glycerol does explain their elevated total carotenoids level compared to cells grown on glucose (Figure 1a). In the genome of RT2316-16, gene sequences for a glycerol-3-phosphate dehydrogenase (Thraus_T878) and two mGPDH (Thraus_T1613 and T1688) have been annotated [15], suggesting that glycerophosphate shuttle may indeed be active in this microorganism.

The lower level of total lipids in the biomass grown on glycerol relative to cells grown on glucose (Figure 1a) under nitrogen sufficiency could plausibly be explained by glycerol inducing the activity of mGPDH for oxidative phosphorylation. An elevated activity of mGPDH should decrease the concentration of G3P, a necessary metabolite for the synthesis of glycerolipids (TAG and phospholipids) [23]. In contrast, under nitrogen deficiency (i.e., after a second feeding with glycerol only in a repeated batch culture; Figure 4b), the total lipid content in RT2316-16 rose to 467 ± 45 mg g^−1^ DW, being nearly 85% higher than in the batch culture (Figure 1a). This increase might be explained by an inability to synthesize protein under nitrogen deficiency, so that resources could be diverted to lipid synthesis.

### 3.3. Yeast Extract Enhanced the Phospholipids Content of the Biomass

Phospholipids occur mainly in cell membranes, and changes in the phospholipid content of the biomass suggest a changing content of membrane-rich organelles such as mitochondria in the cell. The number of mitochondria per cell is known to vary with the stage of the cell cycle [24], and during exponential growth, a higher proportion of the cells may be in a cell cycle phase with a high number of mitochondria per cell [25], leading to the observed changes in the phospholipid content of the biomass. For instance, when the culture was fed once with yeast extract only, a condition that promoted the growth of lipid-free biomass, a high level of phospholipids (25.3 ± 2.7 mg g^−1^ DW) was obtained, while the content of total lipid was reduced by 25% (Figure 2c,d). Because cell growth was promoted, an increased production of phospholipids was required to form the structural membranes, resulting in less of the other lipids.

In oleaginous microorganisms such as thraustochytrids, phospholipids may also form a monolayer coat on the surfaces of lipid droplets [26,27], and this may contribute to an increased phospholipid content of the lipid-rich biomass. Nonetheless, the biomass with increased total lipids (i.e., after the feeding with the carbon source only) contained less phospholipids (Figure 3d and Figure 4d).

The phospholipids in the biomass of RT2316-16 ranged between 15.2 ± 1.5 and 28.7 ± 4.3 mg g^−1^ DW depending on the culture conditions. The best conditions for producing biomass rich in phospholipids occurred after the second feeding with a concentrated CM* containing ascorbic acid (Figure 4). This may have been caused by the mildly growth-promoting effect of ascorbic acid on lipid-free biomass. For the purpose of phospholipid production, a more relevant variable is the phospholipid content in total lipids. The highest levels of phospholipid in total lipids were found in the biomass grown after feeding only the yeast extract (Figure 2).

### 3.4. Carotenoids Synthesis Is Growth Rate Related in RT2316-16

In batch cultures, the total carotenoids in the biomass varied with time, in a similar way to total lipids (Figure 1). After the first day, the synthesis of carotenoids was growth-rate-related until the amino acids were exhausted.

These results suggest that the amino acids provided in yeast extract are used by RT2316-16 for protein synthesis, an energy-intensive process. The required energy is obtained from the reactions in the electron transfer chain in which ROSs are also generated. Carotenoids are synthesized to reduce the damage associated with ROS. The fact that yeast extract induced synthesis of a given carotenoid might be related to its final location, cell membranes or lipid bodies.

β-Carotene is a nonpolar carotenoid that can readily distribute itself in cell membranes and lipid bodies that might exist in a lipid-rich biomass produced via glucose-only feeding (Figure 2c). Unlike β-carotene, canthaxanthin and astaxanthin have polar keto groups at each end of their molecules and, as a consequence, they are embedded in a lipid-bilayer membrane with the polar ends anchored in the opposite polar faces of the bilayer [28].

The polar-end groups in canthaxanthin and astaxanthin can be esterified in the cell to reduce their polarity, allowing their storage in lipid bodies [29]. Furthermore, in some microalgae, the esterification of astaxanthin has been shown to stimulate its synthesis from β-carotene by relieving the feedback inhibition of carotenogenesis that would be caused by the nonesterified product. A similar mechanism may control the synthesis of canthaxanthin in RT2316-16 under certain growth conditions. Should this be so, the culture conditions stimulating lipid synthesis (mainly neutral lipids) would decrease the available fatty acid pool for esterification with polar carotenoids, enabling the inhibition of their synthesis. Thus, the accumulation of nonesterified canthaxanthin or astaxanthin may inhibit their production from β-carotene, allowing the latter to accumulate, as seen in Table 1.

The total carotenoids in RT2316-16 were lower than the values reported for related strains. For example, Park et al. [21] reported that using an optimized growth medium, a temperature-shift and light-emitting diodes, an improved mutant of *Schizochytrium* sp. SHG104 produced 10.8 g L^−1^ of biomass and 4.6 mg L^−1^ of astaxanthin (i.e., 426 mg g^−1^). The results for RT2316-16 suggest that a similar or higher concentration of carotenoids can be obtained if the biomass concentration is increased by using fed-batch operation with a medium containing yeast extract.

The effect of incubation temperature on the total carotenoids in the biomass of RT2316-16 also depended on the composition of the culture medium. Under conditions that favored cell growth (Figure 5), carotenoid accumulation was promoted when the incubation temperature was 15 °C (i.e., when the biomass grew rapidly). This was consistent with the previously noted growth-rate-related synthesis of carotenoids in RT2316-16. On the other hand, a relatively short period (24 h) of storage at 4 °C after the cell had grown at 15 °C increased the content of total carotenoids (Figure 6). In studies with a different thraustochytrid (*Schizochytrium* sp. SHG104), a temperature shift from 28 °C to 20 °C was reported to enhance the carotenoids in the biomass [21]. The increase in total carotenoids in RT2316-16 after a short cold storage was explained as a possible response to cold stress, or an increased availability of one or more precursors of carotenoid synthesis. A possible precursor is farnesyl pyrophosphate (FPP), produced via the mevalonate pathway. The genes for all the relevant enzymes in this pathway were annotated in the genome of RT2316-16 [15]. A combination of nitrogen limitation and the short 4 °C storage (Figure 6) may have caused more FPP to be channeled to carotenoid synthesis instead of the alternative route involving the synthesis of sterols components of the cell membranes via the cholesterol pathway. The latter pathway was likely to have been depressed when the cell did not grow or grew slowly because of the low temperature.

### 3.5. Fatty Acids Produced by RT2316-16 under Different Conditions

During active growth (Figure 1), RT2316-16 always accumulated more saturated fatty acids and monounsaturated fatty acids compared to the sum of EPA and DHA (Table 2 and Table 3). As in thraustochytrids in general, DHA and EPA are ultimately produced at least partly from saturated fatty acid precursors (C16:0, C18:0; [1]). The relatively high concentrations of saturated precursors compared to EPA and DHA (Table 2) and the absence of other intermediate fatty acids (e.g., C18:3, C18:4, C20:4 and C22:5) suggest possible bottlenecks in the fatty acid synthase aerobic pathway [1]. On the other hand, a low level of EPA compared to DHA suggests its rapid conversion to the latter. In addition to the fatty acid synthase aerobic pathway, many thraustochytrids use a second pathway (the polyketide synthase (PKS)-like anaerobic pathway) for making EPA and DHA [1], but this route may not operate in RT2316-16, as an earlier study showed this thraustochytrid to lack the genes for the enzymes involved in the polyketide synthase (PKS)-like pathway [15].

The fatty acid profile of the total lipids in the biomass at different time stages (Table 2) suggests that the synthesis of PUFA was promoted during nitrogen-sufficient growth (first 24 h), whereas saturated and monounsaturated fatty acids were preferentially synthesized under nitrogen deficiency. These results could also be explained by differences in specific growth rate. Considering only the data for day 2 (Table 2, Figure 1), with glucose, the culture under light grew slower than in the dark (Figure 1b), and the lipids produced under light had a higher content of EPA and DHA (Table 2). On the other hand, in the dark, the growth on glycerol was faster than on glucose (Figure 1a), and the lipids from the latter contained more EPA and DHA. Thus, the more rapidly growing cells had less PUFA (EPA + DHA) in their total lipids.

## 4. Materials and Methods

### 4.1. Culture Experiments

#### 4.1.1. Inoculum Preparation 

*Thraustochytrium* sp. RT2316-16 [14] was used in all experiments. Stock cultures were stored in glycerol (50% *v*/*v*) at −18 °C. Experiments were carried out aseptically. The inoculum for all experiments was prepared as follows: A 250 mL Erlenmeyer flask containing 100 mL of sterile control medium (CM) was inoculated with 1 mL of the pure stock culture and incubated on an orbital shaker (150 rpm, 15 °C) in the dark for 5 days. A 5 mL portion of the grown culture was used to inoculate 100 mL of fresh sterile CM. This culture incubated for 5 days, as specified above, was the inoculum for the experiments. The control medium (CM) contained the following components (per L of medium): glucose (Merck KGaA, Darmstadt, Germany) 20 g, yeast extract (Merck) 6 g, monosodium glutamate (Merck) 0.6 g, and half-strength artificial seawater (ASW) [30]. The medium was sterilized by autoclaving (121 °C, 20 min). Filter-sterilized (0.2 μm nominal-pore-size sterile filter) solutions of trace minerals and vitamins were added to the sterile medium [30]. A relatively low incubation temperature (15 °C) was used in the inoculum preparation and subsequent culture, as it had been previously shown to be optimal for a closely related cold-water thraustochytrid [31] also isolated from Antarctic waters.

#### 4.1.2. Effect of the Carbon Source and Light on the Production of Biomass and Lipids 

The growth curve was obtained by culturing the cells in 12 identical Erlenmeyer flasks (250 mL; 100 mL CM per flask) incubated in an orbital shaker (150 rpm, 15 °C) in the dark. Three flasks were withdrawn every 24 h for analysis. The biomass was recovered by centrifugation (2057× *g* 10 min, 4 °C), washed with distilled water, freeze-dried, weighed and stored at −20 °C until further analysis. A portion of the culture supernatant was filtered (0.2 μm nominal-pore-size polytetrafluoroethylene (PTFE) membrane) and frozen (−20 °C) until further analysis.

In a second experiment carried out under the conditions specified above, glycerol (Merck) at 20 g L^−1^ was used to replace glucose as the carbon source. The glycerol-containing medium was designated as CM*.

In a further experiment, 12 culture flasks containing CM were incubated under the conditions specified above but under continuous light (fluorescent lamps, 230 lux).

#### 4.1.3. Repeated-Batch Cultures 

All cultures were grown at 15 °C for 5 days in Erlenmeyer flasks (250 mL, containing 100 mL of sterile medium each) unless specified otherwise. On day 6, each flask was fed with 50 mL of a sterile concentrated feed (first feeding) and incubated for the specified time. In some experiments, the flasks were fed again (second feeding) with 50 mL (per flask) of a sterile feed. All feeds were prepared at a sufficient concentration so that the concentration of glucose and yeast extract in the culture supernatant after the feeding attained the values specified. Culture data before the feeding were obtained by sacrificing three flasks prepared as described above while the other three flasks were fed. After incubation, the biomass in the culture broth was recovered by centrifugation, freeze-dried, weighed and stored at −20 °C until further analysis. A portion of the culture supernatant was filtered (0.2 μm nominal pore size PTFE membrane) and frozen (−20 °C) until further analysis.

In single-feeding experiments, the inoculum preparation and the batch stages used CM. The objective of the feeding was to increase the concentration of the nutrient to the same level as in CM, or the concentration of yeast extract to 6 g L^−1^, or the concentration of glucose to 20 g L^−1^. After feeding, the cultures were incubated for 4 days under the same conditions as in the batch phase, and harvested.

In some experiments, the cultures were fed twice. The objective here was to examine the effects of the concentration of some nutrients after the second feeding. The inoculum preparation and the batch phase used CM. The concentration of the first feed (50 mL) was calculated so that after the feeding the concentrations of the nutrients in the culture broth were the same as in the original CM. Thus, the feed solution (in 1 L of half-strength ASW) contained glucose 60 g, yeast extract 18 g and monosodium glutamate 1.8 g. Five days after the first feeding, groups of three flasks were fed a second time with 50 mL of a sterile feed containing glucose and/or yeast extract in sufficient amounts to bring the concentration of these nutrients in the culture to the level of the original CM or glucose to 20 g L^−1^, or glucose to 20 g L^−1^ and yeast extract to 3 g L^−1^ (designated as CM3), or the level of the original CM and ascorbic acid to 2 g L^−1^. After the second feeding, the broth in the flask was transferred to a 500 mL sterile Erlenmeyer flask. Incubation was continued for 5 days, and the biomass was harvested, freeze-dried, weighed and stored at −20 °C until further analysis. A portion of the culture supernatant was filtered (0.2 μm nominal pore size PTFE membrane) and frozen (−20 °C) for further analysis.

A second culture experiment involving two feeding steps was carried out. All the culture conditions were the same as described above, but glycerol was used as the carbon source instead of glucose; this medium, designated CM*, had all the other nutrients at the same concentration as CM. CM* was used for the inoculum preparation and the batch phase.

A third culture experiment with two feedings was used to examine the effects of the second feed composition and the incubation temperature. In relevant experiments, the culture temperature was readjusted after the second feeding to either 5 °C or 15 °C. The inoculum preparation and the batch phases used CM. The inoculum and batch phases lasted 5 days each. The concentration of the first feed (50 mL) was calculated so that the feeding brought the glucose and yeast extract concentrations in the culture broth to the values in the CM. After 5 days, groups of three flasks were fed again (second feeding) with 50 mL of a sterile concentrated medium such that the nutrients in the broth after the feeding attained the following concentrations (g L^−1^): glucose 30 or glucose 30 + yeast extract 3 + monosodium glutamate 0.3 (i.e., the composition of CM3). The flasks were incubated at 15 °C for 6 days and harvested. A second parallel set of six flasks fed to attain the concentrations specified earlier in this paragraph (same as CM3) was incubated at 5 °C. The biomass in cultures incubated at 15 °C and 5 °C was harvested 6 and 10 days, respectively, after the second feeding.

In a fourth experiment with two feeding steps, the second bolus feed comprised concentrated glucose. The second feed was sufficiently concentrated so that after the feeding the glucose concentration in the culture broth was 20, 30 or 40 g L^−1^. The inoculum preparation and the batch phase for these experiments used CM. The inoculum had been grown for 5 days, and the batch cultures had run for 6 days before the first feeding. The first feed (50 mL) had been formulated to bring the glucose and yeast extract in the fed culture to the same concentrations as in CM. The second feeding occurred 6 days after the first feeding. After the second feeding, the different culture flasks attained glucose concentrations of 20, 30 or 40 g L^−1^. Depending on the glucose concentration, incubation was continued for 6 days (glucose = 20 g L^−1^), 7 days (glucose = 30 g L^−1^) or 8 days (glucose = 40 g L^−1^). At this point, half the volume of each flask was harvested for analysis, and the other half was incubated further at 4 °C for 24 h before harvesting. All the experiments were carried out in triplicate.

### 4.2. Analysis

#### 4.2.1. Concentrations of Biomass and Residual Sugars 

The concentration of biomass (dry weight, DW) was measured gravimetrically by recovering the cells by centrifugation (7000× *g*, 4 °C, 10 min) from a known volume of culture (~10 mL). The cell pellet was washed twice with distilled water (5 mL per wash), recovered by centrifugation, and dried (65 °C) to constant weight.

Concentrations of residual glucose and glycerol were measured by high-performance liquid chromatography (HPLC) (Alliance Waters e2695 Separation Module; Waters Inc, Milford, MA, USA). A sugar HPX-87H column (Bio-Rad Laboratories Inc., Hercules, CA, USA) held at 65 °C was used for glucose measurements. The mobile phase was sulfuric acid (5 mM) at a flow rate of 0.6 mL min^−1^. A Shodex KS-800 (Showa Denko, Tokyo, Japan) column held at 80 °C was used for glycerol measurements. The mobile phase was deionized water at a flow rate of 1 mL min^−1^. A refractive index (Waters Inc., Milford, MA, USA) detector was used for both analytes.

#### 4.2.2. Extraction of Total Lipids and Determination of Fatty Acid Profile 

Total lipids (TL) in the biomass were extracted using the Bligh and Dyer method [32]. A 50 mg portion of the freeze-dried biomass was extracted (1 h, 150 rpm) with 9.5 mL of a solvent mixture of chloroform: methanol: phosphate buffer (50 mM, pH 7.4) 2.5: 5.0: 2.0 by volume. This slurry was transferred to a separating funnel containing 2.5 mL of chloroform. After mixing, 2.5 mL of phosphate buffer was added, and the contents were mixed and allowed to separate. The chloroform layer was recovered, the solvent was evaporated at room temperature and the residue of the extracted TL was weighed.

The extracted lipids were methylated (2 M KOH in methanol) and further extracted into petroleum ether. The ratio of alkaline methanol to petroleum ether in the methylation-extraction system was 1: 10 by volume. The reaction system was thoroughly mixed (2 min) at room temperature and allowed to stand for 1 h. The petroleum ether layer was recovered by centrifugation (10,000× *g*, 4 °C, 5 min) and evaporated at room temperature in a fume hood to recover the fatty acid methyl esters (FAME).

The FAME profile was determined using a gas chromatograph (GC-2010 Plus; Shimadzu, Kyoto, Japan) equipped with a flame ionization detector and a split injector. A fused silica capillary column (Rtx-2330; 60 m × 0.32 mm × 0.2 μm film thickness; Thames Restek, Saunderton, UK) was used. Nitrogen was the carrier gas. The column temperature profile was as follows: 140 °C for 5 min, then increased to 240 °C at 3 °C min^−1^ and held at this temperature for 5 min. The injector and the detector were held at 260 °C, and FAME was identified with reference to a 37-component standard FAME Mix (Supelco, Bellefonte, PA, USA). Individual fatty acids were reported as the percentage of the total fatty acids in the TL.

#### 4.2.3. Quantification of Phospholipids 

The phospholipid (PL) content in total lipid (TL) fraction was estimated by measuring the phosphorous in TL extracted as described in the previous section. The total phosphorus was determined using the method Ca 12-55 [33] adapted to a small volume. Briefly, the TL sample (400 μL) was vortex-mixed with 400 μL sulfuric acid (5 M) in a glass tube, incubated in an oven (200 °C, 1 h) and then cooled to room temperature. Hydrogen peroxide (100 μL) was added, and the mixture was further incubated (200 °C, 1.5 h). The mixture was cooled to room temperature, and 4.6 mL of ammonium heptamolybdate solution (made by dissolving 1.1 g of ammonium heptamolybdate in 12.5 mL sulfuric acid (5 M) diluted with 100 mL distilled water) was added. The content of the tube was mixed, and 100 μL of a 15% *w*/*v* ascorbic acid solution (in distilled water) was added. This mixture was incubated at 100 °C for 7 min and cooled to room temperature, and its spectrophotometric absorbance was read at 830 nm. The measured absorbance was converted to the quantity of phosphorus (*m*_P_, in mg) using a calibration curve that had been made using a standard solution of monobasic potassium phosphate treated the same way as the sample. The PL content in the dry biomass was calculated using the following equation:

(1) $$\mathrm{PL}\left(\frac{\mathrm{mg}}{g\;\mathrm{DW}}\right)=\frac{m_{P}}{m_{B}}\;$$

In the above equation, *m*_B_ (g) was the quantity of dry biomass used in extracting the TL.

#### 4.2.4. Extraction and Quantification of Carotenoids 

A culture aliquot (3–5 mL) was centrifuged (2057× *g*, 10 min), and the supernatant was discarded. The cell pellet was suspended in 1 mL of a salt solution (300 mM NaCl in 50 mM phosphate buffer, pH 8.0), vortex-mixed for 30 s and then sonicated for 20 min (E60H Elmasonic sonicator; Elma Schmidbauer GmbH, Singen, Germany). The suspension was centrifuged to recover the solids. The solids were extracted with 1 mL of a methanol: chloroform mixture (2:1 *v*/*v*) for 1 h while being continuously mixed on a vortex mixer (ZX3; Velp Scientifica, Usmate Velate, Italy). The supernatant was recovered by centrifugation. The pellet was extracted repeatedly, as above, until the solid residue became white. Spectrophotometric absorbance of the pooled extract was measured at 460 nm (*A*_460_) against a blank of methanol: chloroform (2:1 *v*/*v*) [34]. Total carotenoid (TC) content of dry biomass was calculated using the following equation:

(2) $$\mathrm{TC}\left(\frac{\mu g}{g\;\mathrm{DW}}\right)=\frac{5.405\;A_{460}V}{X}$$

In the above equation, *V* was the volume (mL) of the pooled extract, *X* (g) was the quantity of dry biomass extracted and 5.405 was the average molar extinction coefficient of the following ten carotenoids: lycopene, α-carotene, β-carotene, γ-carotene, zeaxanthin, rhodoxanthin, astaxanthin, lutein, α-apo-2-carotenal and dihydro-α-carotene.

Individual carotenoids (cantaxanthin, astaxanthin, β-carotene) in the biomass were quantified following a suitably adapted published method [35]. Briefly, 50 mg of the dry biomass was mixed with 1 mL of a chloroform: methanol solution (1:2 *v*/*v*) and extracted by continuously mixing (vortex mixer) for 20 min. The supernatant was recovered by centrifugation (10,000× *g*, 20 min), and the solvent was evaporated. The recovered pigments were saponified by suspending the residue in 500 μL of the above-mentioned chloroform: methanol solution, mixing with 50 μL of a KOH solution (2 M in methanol), followed by incubation at 40 °C for 30 min. The liquid phase was then recovered by centrifugation (10,000× *g*, 10 min) and analyzed by HPLC. A Symmetry C18 (4.6 × 250 mm, 5 μm, Waters) column held at 30 °C was used. The mobile phase consisted of a solvent system of acetonitrile: methanol: tetrahydrofuran in the volume ratio of 70:25:5. The flow rate of the mobile phase was 1 mL min^−1^. Carotenoids were detected by measuring the absorbance at 445 nm. Solutions of authentic canthaxanthix, astaxanthin and β-carotene (Merck) were used for calibration.

#### 4.2.5. Statistical Analysis 

MATLAB (MathWorks, Inc., Natick, MA, USA) was used to perform one-way analysis of variance (ANOVA) and comparison of the means at a 95% confidence level.

## 5. Conclusions

The composition of *Thraustochytrium* sp. RT2316-16 biomass strongly depended on the composition of the culture medium. Light and biotin had no effect on the content of phospholipids and carotenoids, and the relatively minor effect of ascorbic acid depended on the carbon source. The most significant effect was that of yeast extract, a source of preformed amino acids. Yeast extract promoted cell growth and increased the concentration of the lipid-free biomass. This biomass had a high content of carotenoids, mainly canthaxanthin, and phospholipids in the total lipids, and potentially more EPA and DHA. In a medium deprived of yeast extract, the RT2316-16 biomass had a low content of carotenoids, mainly β-carotene, but was enriched in lipids (>40% *w*/*w*), especially if glycerol was the carbon source. The lipids synthesized under these conditions had a low content of phospholipids and a high content of saturated and monounsaturated fatty acids.

The significant contribution of the amino acids to the production of lipids rich in EPA and DHA, and the oxygenated carotenoids such as canthaxanthin, suggests a need to either identify inexpensive sources of amino acids to replace yeast extract or reduce its concentration. In practice, high-density heterotrophic fed-batch culture of *Thraustochytrium* sp. has the potential for attaining higher productivities of carotenoids than is possible with the relatively low-density photoautotrophic cultures of microalgae.

## Figures and Tables

**Table 1:** Effect of culture conditions on carotenoids production and composition in RT2316-16.

Culture Conditions	Total Carotenoids (μg g^−1^)	Canthaxanthin (%)	Astaxanthin (%)	β-Carotene (%)
Batch * (glucose in the dark)	37 ± 4	86.4 ± 0.4	5.4 ± 0.9	8.7 ± 2.0 ^b^
Batch * (glucose with light)	33 ± 5	76.5 ± 4.2 ^a^	5.8 ± 1.0	17.7 ± 3.1 ^a^
Batch * (glycerol in the dark)	55 ± 5 ^a^	87.4 ± 0.7	7.1 ± 0.4	5.5 ± 0.3 ^c^
Repeated-batch ^⁑^ (fed with CM)	152 ± 9 ^b^	57.9 ± 3.7 ^a^	0.0 ± 0.0	40.8 ± 1.8 ^b^
Repeated-batch ^⁑^ (fed with YE)	168 ± 7 ^a^	61.4 ± 0.2 ^a^	5.1 ± 1.0 ^a^	33.4 ± 1.1 ^c^
Repeated-batch ^⁑^ (fed with glucose)	43 ± 3 ^c^	5.7 ± 2.0 ^b^	0.0 ± 0.0	95.6 ± 2.7 ^a^

* Biomass for analysis was harvested on day 4 after inoculation. ^⁑^ Cells were grown for 4 days in the control medium (CM). On day 6, the culture was fed with a concentrated solution containing either yeast extract only (YE), glucose only, or CM. The biomass for analysis was harvested 4 days after the feeding. A different superscript letter within a column block (Batch or Repeated-batch experiments) indicates a significant difference at *p* < 0.05.

**Table 2:** Effect of incubation period (days) on the fatty acid (% by wt) profile of the total lipids in the biomass of RT2316-16 grown at 15 °C in batch culture using glucose, or glycerol, with or without light.

Fatty Acid	Glycerol, Dark			Glucose, DARK			Glucose, Light		
	Day 2	Day 3	Day 4	Day 2	Day 3	Day 4	Day 2	Day 3	Day 4
C14:0	4.0 ± 0.2	5.0 ± 0.2	5.2 ± 0.1	3.5 ± 0.4	5.6 ± 0.2	5.4 ± 0.2	0.0 ± 0.0	5.0 ± 0.3	5.3 ± 0.3
C16:0	22.5 ± 0.1	21.4 ± 0.7	21.8 ± 0.5	31.2 ± 6.6	28.8 ± 1.0	26.9 ± 0.7	30.1 ± 0.4	26.6 ± 2.5	28.0 ± 1.1
C16:1	0.0 ± 0.0	1.6 ± 0.0	1.4 ± 0.6	0.0 ± 0.0	1.7 ± 0.2	2.4 ± 0.3	0.0 ± 0.0	1.2 ± 0.1	1.7 ± 0.1
C18:0	15.1 ± 0.2	23.2 ± 1.5	21.3 ± 1.4	12.9 ± 2.8	21.8 ± 0.6	13.3 ± 0.2	9.4 ± 1.2	18.0 ± 2.8	16.9 ± 4.3
C18:1cis	33.0 ± 1.9	35.5 ± 1.8	41.4 ± 0.4	23.4 ± 5.2	29.9 ± 2.9	41.0 ± 1.2	17.9 ± 1.0	29.4 ± 0.5	36.5 ± 1.0
C18:2cis	5.1 ± 0.1	4.4 ± 0.3	3.6 ± 0.4	4.7 ± 0.7	5.5 ± 0.9	5.7 ± 0.8	5.9 ± 1.4	5.9 ± 1.4	8.8 ± 0.6
C22:1	1.9 ± 0.1	1.4 ± 0.3	1.2 ± 0.1	0.0 ± 0.0	1.1 ± 0.2	0.6 ± 0.5	0.0 ± 0.0	0.0 ± 0.0	0.0 ± 0.0
C20:5n-3 (EPA)	3.9 ± 0.3	1.8 ± 0.3	1.3 ± 0.6	8.8 ± 0.9	1.7 ± 0.2	2.2 ± 0.9	13.0 ± 3.9	4.8 ± 0.3	1.7 ± 0.3
C24:1	1.9 ± 1.6	0.0 ± 0.0	0.0 ± 0.0	0.0 ± 0.0	0.0 ± 0.0	0.0 ± 0.0	0.0 ± 0.0	0.0 ± 0.0	0.0 ± 0.0
C22:6n-3 (DHA)	12.6 ± 1.0	5.6 ± 1.1	2.5 ± 0.4	19.6 ± 3.3	3.8 ± 2.2	2.6 ± 0.8	29.6 ± 1.3	7.9 ± 0.4	4.4 ± 1.3
Others	5.1	4.4	4.1	0.6	5.5	4.8	0.0	7.2	6.1

**Table 3:** Effect of incubation temperature and the composition of the second feed (CM3, or glucose only) on the fatty acid (% by wt) profile of total lipids in the biomass of RT2316-16 grown in repeated-batch culture at two different temperatures *.

Fatty Acid	CM3		Glucose	
	5 °C	15 °C	5 °C	15 °C
C14:0	6.6 ± 0.5	3.4 ± 0.2	12.5 ± 7.4	5.1 ± 0.9
C15:0	4.7 ± 0.3	2.0 ± 0.0	0.0 ± 0.0	0.0 ± 0.0
C16:0	29.3 ± 1.0	17.0 ± 0.6	21.0 ± 1.6	23.4 ± 7.6
C16:1	3.2 ± 0.5	1.7 ± 0.1	13.9 ± 1.6	9.0 ± 5.2
C17:0	1.2 ± 0.0	2.7 ± 0.1	0.0 ± 0.0	0.0 ± 0.0
C18:0	28.3 ± 0.3	25.4 ± 0.7	12.6 ± 1.1	23.0 ± 8.2
C18:1cis	18.7 ± 1.1	34.2 ± 0.6	19.9 ± 3.3	29.4 ± 3.9
C18:2cis	1.6 ± 0.1	0.4 ± 0.3	3.8 ± 0.4	3.1 ± 1.9
C18:3cis6,9,12	1.2 ± 0.0	0.0 ± 0.0	4.5 ± 0.3	0.0 ± 0.0
C24:0	0.0 ± 0.0	1.8 ± 0.1	0.0 ± 0.0	1.4 ± 0.1
C20:5n-3 (EPA)	1.4 ± 0.1	1.6 ± 0.2	4.9 ± 0.1	1.1 ± 1.0
C24:1	0.0 ± 0.0	3.4 ± 0.7	0.0 ± 0.0	2.4 ± 1.6
C22:6n-3 (DHA)	2.3 ± 0.2	5.6 ± 1.0	6.6 ± 0.5	2.1 ± 1.7
Others	1.4	0.8	0.4	0.0 ± 0.0

* Incubation temperature prior to second feeding was always 15 °C. The data shown are for specified incubation temperatures after the second feeding.

## Supplementary Materials

The following supporting information can be downloaded at: https://www.mdpi.com/article/10.3390/md20070416/s1. The Excel file contains the experimental data shown in Figures S1 to S6 [36].

## References

[1] Chi G., Xu Y., Cao X., Li Z., Cao M., Chisti Y., He H. (2022). Production of polyunsaturated fatty acids by *Schizochytrium* (*Aurantiochytrium*) spp.. Biotechnol. Adv..

[2] Abe E., Hayashi Y., Hama Y., Hayashi M., Inagaki M., Ito M. (2006). A novel phosphatidylcholine which contains pentadecanoic acid at sn-1 and docosahexaenoic acid at sn-2 in *Schizochytrium* sp. F26-b. J. Biochem..

[3] Abe E., Ikeda K., Nutahara E., Hayashi M., Yamashita A., Taguchi R., Doi K., Honda D., Okino N., Ito M. (2014). Novel lysophospholipid acyltransferase PLAT1 of *Aurantiochytrium limacinum* F26-b responsible for generation of palmitate-docosahexaenoate-phosphatidylcholine and phosphatidylethanolamine. PLoS ONE.

[4] Morita E., Kumon Y., Nakahara T., Kagiwada S., Noguchi T. (2006). Docosahexaenoic acid production and lipid-body formation in *Schizochytrium limacinum* SR21. Mar. Biotechnol..

[5] Yue X.H., Chen W.C., Wang Z.M., Liu P.Y., Li X.Y., Lin C.B., Lu S.H., Huang F.H., Wan X. (2019). Lipid Distribution pattern and transcriptomic insights revealed the potential mechanism of docosahexaenoic acid traffics in *Schizochytrium* sp. A-2. J. Agric. Food Chem..

[6] Chodchoey K., Verduyn C. (2012). Growth, fatty acid profile in major lipid classes and lipid fluidity of *Aurantiochytrium mangrovei* SK-02 As a function of growth temperature. Braz. J. Microbiol..

[7] Okuyama H., Orikasa Y., Nishida T. (2007). In vivo conversion of triacylglycerol to docosahexaenoic acid-containing phospholipids in a thraustochytrid-like microorganism, strain 12B. Biotechnol. Lett..

[8] Chang G., Luo Z., Gu S., Wu Q., Chang M., Wang X. (2013). Fatty acid shifts and metabolic activity changes of *Schizochytrium* sp. S31 cultured on glycerol. Bioresour. Technol..

[9] Ren L.J., Sun G.N., Ji X.J., Hu X.C., Huang H. (2014). Compositional shift in lipid fractions during lipid accumulation and turnover in *Schizochytrium* sp.. Bioresour. Technol..

[10] Lagarde M., Bernoud N., Brossard N., Lemaitre-Delaunay D., Thies F., Croset M., Lecerf J. (2001). Lysophosphatidylcholine as a preferred carrier form of docosahexaenoic acid to the brain. J. Mol. Neurosci..

[11] Picq M., Chen P., Perez M., Michaud M., Vericel E., Guichardant M., Lagarde M. (2010). DHA metabolism: Targeting the brain and lipoxygenation. Mol. Neurobiol..

[12] Aki T., Hachida K., Yoshinaga M., Katai Y., Yamasaki T., Kawamoto S., Kakizono T., Maoka T., Shigeta S., Suzuki O. (2003). Thraustochytrid as a potential source of carotenoids. J. Am. Oil Chem. Soc..

[13] Britton G. (1995). Structure and properties of carotenoids in relation to function. FASEB J..

[14] Galasso C., Corinaldesi C., Sansone C. (2017). Carotenoids from marine organisms: Biological functions and industrial applications. Antioxidants.

[15] Leyton A., Flores L., Shene C., Chisti Y., Larama G., Asenjo J.A., Armenta R.E. (2021). Antarctic thraustochytrids as sources of carotenoids and high-value fatty acids. Mar. Drugs.

[16] Tong L. (2013). Structure and function of biotin-dependent carboxylases. Cell. Mol. Life Sci..

[17] Ren L., Sun X., Ji X., Chen S., Guo D., Huang H. (2017). Enhancement of docosahexaenoic acid synthesis by manipulation of antioxidant capacity and prevention of oxidative damage in *Schizochytrium* sp.. Bioresour. Technol..

[18] Zhang S., Chen X., Sen B., Bai M., He Y., Wang G. (2021). Exogenous antioxidants improve the accumulation of saturated and polyunsaturated fatty acids in *Schizochytrium* sp. PKU#Mn4. Mar. Drugs.

[19] Gupta A., Barrow C.J., Puri M. (2022). Multiproduct biorefinery from marine thraustochytrids towards a circular bioeconomy. Trends Biotechnol..

[20] Schöpf L., Mautz J., Sandmann G. (2013). Multiple ketolases involved in light regulation of canthaxanthin biosynthesis in *Nostoc punctiforme* PCC 73102. Planta.

[21] Park H., Kwak M., Seo J., Ju J., Heo S., Park S., Hong W. (2018). Enhanced production of carotenoids using a Thraustochytrid microalgal strain containing high levels of docosahexaenoic acid-rich oil. Bioprocess Biosyst. Eng..

[22] Orr A.L., Quinlan C.L., Perevoshchikova I.V., Brand M.D. (2012). A refined analysis of superoxide production by mitochondrial sn-glycerol 3-phosphate dehydrogenase. J. Biol. Chem..

[23] Mráček T., Drahota Z., Houštěk J. (2013). The function and the role of the mitochondrial glycerol-3-phosphate dehydrogenase in mammalian tissues. Biochim. Biophys. Acta.

[24] Cole L.W. (2016). The evolution of per-cell organelle number. Front. Cell Dev. Biol..

[25] Goranov A.I., Cook M., Ricicova M., Ben-Ari G., Gonzalez C., Hansen C., Tyers M., Amon A. (2009). The rate of cell growth is governed by cell cycle stage. Genes Dev..

[26] Fei W., Zhong L., Ta M.T., Shui G., Wenk M.R., Yang H. (2011). The size and phospholipid composition of lipid droplets can influence their proteome. Biochem. Biophys. Res. Commun..

[27] Tauchi-Sato K., Ozeki S., Houjou T., Taguchi R., Fujimoto T. (2002). The surface of lipid droplets is a phospholipid monolayer with a unique fatty acid composition. J. Biol. Chem..

[28] Gruszecki W.I., Strzałka K. (2005). Carotenoids as modulators of lipid membrane physical properties. Biochim. Biophys. Acta Mol. Basis Dis..

[29] Chen G., Wang B., Han D., Sommerfeld M., Lu Y., Chen F., Hu Q. (2015). Molecular mechanisms of the coordination between astaxanthin and fatty acid biosynthesis in *Haematococcus pluvialis* (Chlorophyceae). Plant J..

[30] Shene C., Leyton A., Rubilar M., Pinelo M., Acevedo F., Morales E. (2013). Production of lipids and docosahexaenoic acid (DHA) by a native *Thraustochytrium* strain. Eur. J. Lipid Sci. Technol..

[31] Paredes P., Larama G., Flores L., Leyton A., Ili C.G., Asenjo J.A., Chisti Y., Shene C. (2020). Temperature differentially affects gene expression in Antarctic thraustochytrid *Oblongichytrium* sp. RT2316-13. Mar. Drugs.

[32] Bligh E., Dyer W. (1959). A rapid method for total lipid extraction and purification. Can. J. Biochem. Physiol..

[33] Fire-stone D., AOCS (1993). Official Methods and Recommended Practices of the American Oil Chemists’ Society.

[34] Vijayalakshmi G., Shobha B., Vanajakshi V., Divakar S., Manohar B. (2001). Response surface methodology for optimization of growth parameters for the production of carotenoids by a mutant strain of *Rhodotorula gracilis*. Eur. Food Res. Technol..

[35] Sathish T., Udayakiran D., Himabindu K., Lakshmi P., Sridevi D., Devarapalli K., Bhojaraju P. (2009). HPLC Method for the determination of lycopene in crude oleoresin extracts. Asian J..

[36] Bertrand-Harb C., Nicolas M.G., Dalgalarraondo M., Chobert J.M. (1993). Determination of alkylation degree by three colori-metric methods and amino-acid analysis: A comparative study. Sci. Aliment..

